# Phosphoproteome Analysis Reveals the Molecular Mechanisms Underlying Deoxynivalenol-Induced Intestinal Toxicity in IPEC-J2 Cells

**DOI:** 10.3390/toxins8100270

**Published:** 2016-09-22

**Authors:** Zhi-Qi Zhang, Song-Bo Wang, Rui-Guo Wang, Wei Zhang, Pei-Long Wang, Xiao-Ou Su

**Affiliations:** 1 Institute of Quality Standards and Testing Technology for Agricultural Products, Chinese Academy of Agricultural Science, Key Laboratory of Agrifood Safety and Quality, Ministry of Agriculture, Beijing 100081, China; zzq19830619@163.com (Z.-Q.Z.); wangruiguo@caas.cn (R.-G.W.); zhangwei03@caas.cn (W.Z.); wangpeilong@caas.cn (P.-L.W.); 2 College of Animal Science and National Engineering Research Center for Breeding Swine Industry, South China Agricultural University, Guangzhou 510642, China; songbowang@scau.edu.cn

**Keywords:** deoxynivalenol, intestinal toxicity, phosphoproteomics, signaling pathways

## Abstract

Deoxynivalenol (DON) is a widespread trichothecene mycotoxin that commonly contaminates cereal crops and has various toxic effects in animals and humans. DON primarily targets the gastrointestinal tract, the first barrier against ingested food contaminants. In this study, an isobaric tag for relative and absolute quantitation (iTRAQ)-based phosphoproteomic approach was employed to elucidate the molecular mechanisms underlying DON-mediated intestinal toxicity in porcine epithelial cells (IPEC-J2) exposed to 20 μM DON for 60 min. There were 4153 unique phosphopeptides, representing 389 phosphorylation sites, detected in 1821 phosphoproteins. We found that 289 phosphopeptides corresponding to 255 phosphoproteins were differentially phosphorylated in response to DON. Comprehensive Gene Ontology (GO) analysis combined with Kyoto Encyclopedia of Genes and Genomes (KEGG) pathway enrichment revealed that, in addition to previously well-characterized mitogen-activated protein kinase (MAPK) signaling, DON exposure altered phosphatidylinositol 3-kinase/Akt (PI3K/Akt) and Janus kinase/signal transducer, and activator of transcription (JAK/STAT) pathways. These pathways are involved in a wide range of biological processes, including apoptosis, the intestinal barrier, intestinal inflammation, and the intestinal absorption of glucose. DON-induced changes are likely to contribute to the intestinal dysfunction. Overall, identification of relevant signaling pathways yielded new insights into the molecular mechanisms underlying DON-induced intestinal toxicity, and might help in the development of improved mechanism-based risk assessments in animals and humans.

## 1. Introduction

Deoxynivalenol (DON), also known as vomitoxin, is among the mycotoxins most frequently encountered in cereal-based foods throughout the world [1,2,3]. DON has become an issue of major concern because it elicits toxic effects in humans as well as in animals [4,5]. Among various animal species, the pig is the most affected by DON and is considered the most relevant animal model of human sensitivity [6,7,8,9]. The gastrointestinal tract represents the first barrier against contaminated food and feed [10]. Thus, intestinal epithelial cells (IECs) can be exposed to and thus rapidly absorb a high concentration of DON after ingestion of contaminated food or feed [11,12]. Numerous in vitro and in vivo studies have confirmed that this compound affects IEC function by modulating the proliferation and viability of cells and impairing intestinal barrier functions [12,13,14]. In addition, emerging evidence suggests that DON is directly involved in the inflammation of the intestine [9,15] and inhibits the absorption of nutrients via this organ [16]. It has been reported that phosphorylation of mitogen-activated protein kinases (MAPKs) plays a crucial role in the regulation of downstream events [7,12]. However, the signaling networks involved in DON-mediated biological processes are not well understood. Thus, an integrative approach to elucidating the molecular events evoked by DON in IECs is urgently needed. Recently, mass spectrometry (MS)-based phosphoproteomic methods allowing large-scale identification and quantification of phosphoproteins have been employed to uncover specific signaling events in diverse biological processes [17]. The changes in the proteome and phosphoproteome caused by DON have been previously examined in macrophages, and the immunotoxicity of this compound is well documented [18,19]. However, to the best of our knowledge, there are no proteomic studies investigating the intestinal toxicity mechanisms of DON at the cellular level. An isobaric tag for relative and absolute quantitation (iTRAQ)-based proteomics has been successfully used for accurate characterization and quantitation of changes in global protein expression [20]. Results suggest that this strategy should be ideal for the study of DON-induced phosphoproteomic changes in IECs. 

Our aim was to obtain a thorough molecular perspective on the signaling associated with key biological processes affected by DON, including apoptosis, the intestinal barrier, intestinal inflammation, and the absorption of nutrients. We employed iTRAQ combined with liquid chromatography (LC-MS)/MS to identify and quantitate phosphorproteomic changes in the intestinal porcine epithelial cells (IPEC-J2) exposed to DON (20 μM) for 60 min based on the pharmacokinetic distribution of DON in small intestine [21,22]. IPEC-J2 cells originate from jejunum and retain most of their original epithelial nature [23,24]. They have been used extensively to investigate the molecular mechanisms of DON action [8,13]. Using a comprehensive phosphorproteomic analysis, we found that the DON-induced intestinal toxicity mechanism is particularly intricate, extending far beyond its previously known ability to activate MAPKs.

## 2. Results

### 2.1. Deoxynivalenol (DON) Impaired the Barrier Function of Intestinal Porcine Epithelial Cells (IPEC-J2) 

**Figure 1:**
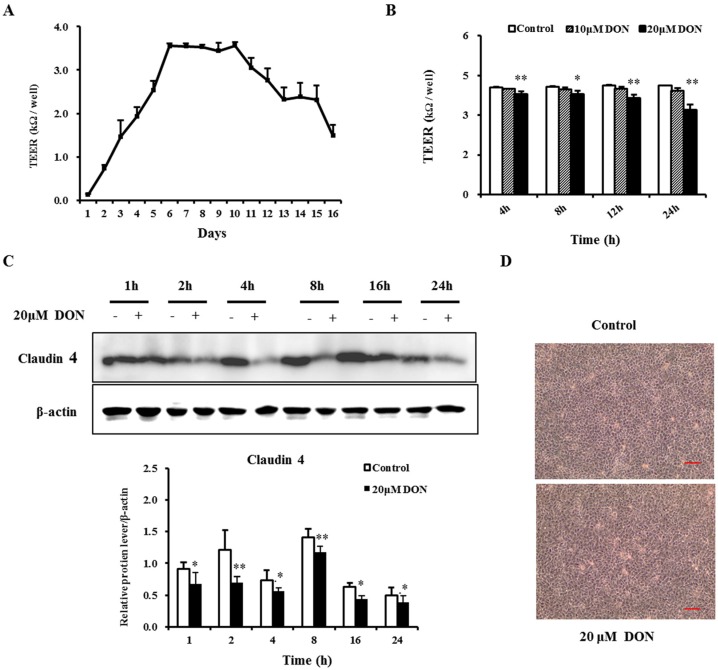
Deoxynivalenol (DON) impaired TEER (transepithelial electrical resistance) and decreased the expression of the claudin-4 protein in differentiated IPEC-J2 cells. Cultured cells reached confluence within two days, and the differentiation medium was then used (changed every other day). (**A**) Changes in TEER in differentiated IPEC-J2 cells were measured during the next 16 days of culture; (**B**) TEER of differentiated IPEC-J2 cells on day 7 after a 4 h, 8 h, 12 h, and 24 h exposure to 10 μM and 20 μM DON; (**C**) Expression of claudin-4 in the differentiated IPEC-J2 cells on day 7 after 1 h, 2 h, 4 h, 8 h, 16 h, and 24 h treatment with 20 μM DON, with β-actin as loading control. Results were obtained using six replicates (*n* = 6). Data are presented as means ± SEM. *, *p* ≤ 0.05; **, *p* ≤ 0.01; (**D**) Cell morphology after 20 μM DON treatment for 24 h. Scale bars in all pictures are 50 μm. Magnification: 100×.

The transepithelial electrical resistance (TEER), an important parameter of barrier function, was measured to evaluate the potential toxic effect of DON on the integrity of the epithelial barrier. Figure 1A shows a continuous increase in TEER in the first five days as a result of parallel formation of a confluent cell layer. Subsequently, TEER reached a maximum of above 3 kΩ/well on day 6, maintained a similar level for the next five days, and then gradually decreased until day 16 (Figure 1A). Thus, on day 7, IPEC-J2 cells were still undergoing differentiation, and TEER was stable. Hence, differentiated IPEC-J2 cells treated with DON on day 7 were chosen for the following analysis. 

After treatment with 20 μM DON over a 24 h period, TEER significantly decreased at 4 h, 8 h, 12 h, and 24 h in a time- and dose-dependent manner (Figure 1B). However, 10 μM DON treatment caused a tendency to reduce TEER, as this effect was not significant. These results suggest that DON likely damages the intestinal barrier and increases the paracellular permeability. 

### 2.2. DON Decreased the Expression of Tight Junction Proteins

To further validate the effects of DON on barrier function, we examined the protein expression of claudin-4. As shown in Figure 1C, after 20 μM DON exposure, there was a significant reduction of the expression in claudin-4 at different time points. Consistent with the molecular level results, DON elicited some morphological changes, as cell connections were loosened and widened (Figure 1D). These data further confirmed that DON treatment caused deterioration in barrier function.

### 2.3. The Role of Apoptosis in DON Toxicity

**Figure 2:**
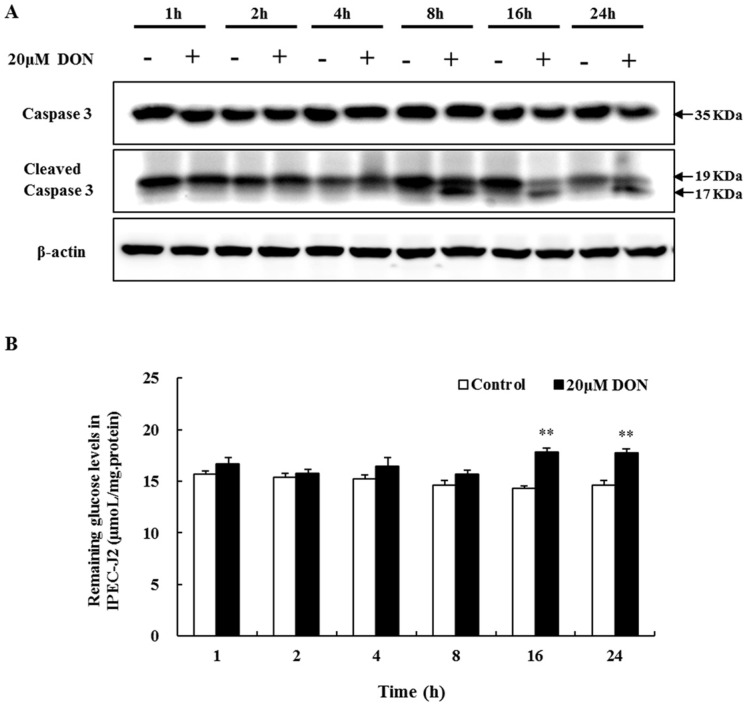
The effects of DON exposure on caspase-3 activity and glucose consumption in differentiated IPEC-J2 cells. (**A**) Caspase-3 and the cleaved caspase-3 fragment from differentiated IPEC-J2 cells on day 7, after 1 h, 2 h, 4 h, 8 h, 16 h, and 24 h treatment with 20 µM DON analyzed with Western blotting, with β-actin as loading control; (**B**) Remaining glucose levels were measured after DON exposure (as described in **A**) and normalized using total protein content. Six replicates were used (*n* = 6), and data are presented as means ± SEM. **, *p* ≤ 0.01.

Caspase-3 is a critical executioner in the induction of apoptosis. The activation of caspase-3 involves its cleavage at Asp175. Here, we analyzed DON-mediated apoptotic effects by examining caspase-3 activity. As expected, after 8 h of treatment with 20 μM DON, the cleaved caspase-3 fragment (19/17 kDa) was detected, whereas it was not observed in the control cells (Figure 2A). These data confirm the DON-triggered apoptosis.

### 2.4. The Effect of DON on Glucose Consumption

Impairment of the intestinal barrier function might be associated with poor nutrient uptake. Glucose consumption assays showed that the amounts of glucose remaining after 1 h, 2 h, 4 h, and 8 h 20 μM DON treatment showed a rising trend, increasing dramatically at 16 h and 24 h (Figure 2B). Thus, in spite of time-course effects not occurring, a relatively long time was needed for a significant inhibition of glucose consumption by DON.

### 2.5. Identification of Phosphopeptide and Phosphoprotein

**Figure 3:**
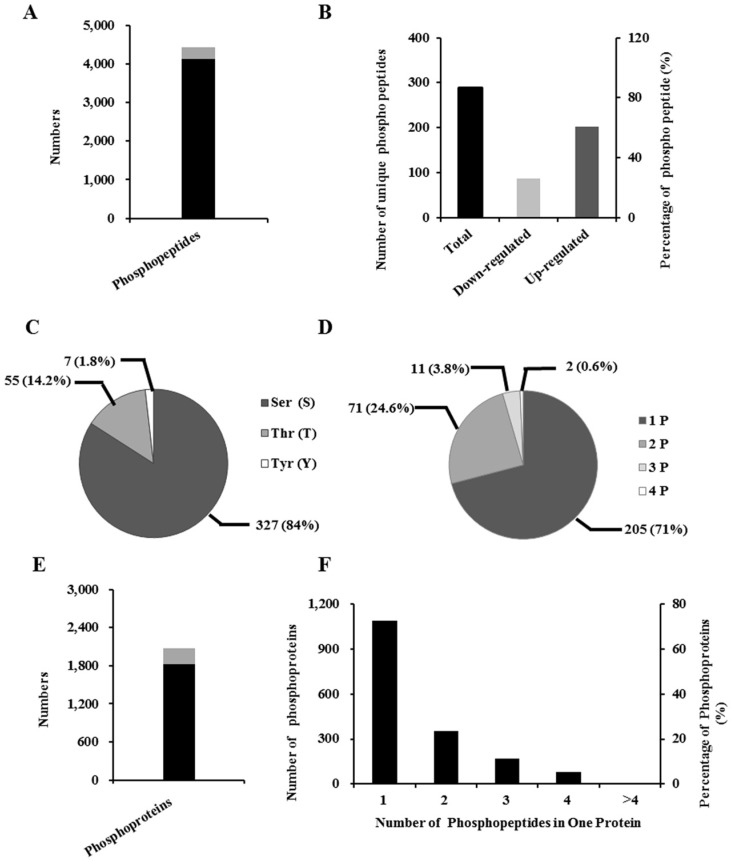
Characterization of phosphorylated peptides, phosphorylation sites, and phosphoproteins in differentiated IPEC-J2 cells after DON exposure. (**A**) The number of differentially regulated phosphorylated peptides (gray) (289) among all the peptides (4134) identified (gray + black), as determined by a fold-change > ±1.2; (**B**) Analysis of the 289 differentially phosphorylated peptides after DON exposure; (**C**) Overall distribution of the Ser, Thr, and Tyr phosphorylation sites; (**D**) Distribution of singly, doubly, triply, and quadruply phosphorylated peptides; (**E**) The number of differentially regulated proteins (gray) (255) among all the phosphorylated proteins (1821) identified (gray + black), as determined by a fold-change > ±1.2; (**F**) Numbers and percentages are based on different numbers of phosphopeptides identified.

Taking into account intestinal impairment, we conducted a phosphoproteomic analysis of IPEC-J2 cells (incubated with and without DON) of DON action. We identified approximately 4134 unique phosphopeptides (Figure 3A and Table S1), and subsequently, on the basis of the criteria of a fold-change > ±1.2, we identified 289 unique phosphopeptides, containing 389 phosphorylation sites (Figure 3A). Among these peptides, 202 (70%) showed an increase, and 87 (30%) a decrease in abundance (Figure 3B and Table S2). Among the detected phosphorylation sites, 327 (84%) were phosphorylated at the serine residues, 55 (14%) at threonine, and seven (2%) at tyrosine (Figure 3C). Among the 289 unique phosphopeptides, 205 were singly phosphorylated, 71 were doubly phosphorylated, 11 were phosphorylated at three sites, and in two cases the peptide was phosphorylated at four sites (Figure 3D).

To identify related proteins, these phosphopeptides were individually searched against the UniProtKB database. For the 4234 unique phosphopeptides, 1821 phosphoproteins were found (Figure 3E and Table S3). On the basis of the criterion of a fold-change > ±1.2, 255 differentially phosphoproteins were identified (Figure 3E). After many stages of protein digestion, phosphopeptide enrichment, fractionation and MS analysis, it seems that two or more phosphopeptides for a single protein were identified. Similarly, our results indicated that, of the 1821 phosphoproteins, 59.82%, 19.48%, 9.33%, 4.39%, and 6.97% were represented by one, two, three, four or more than four phosphopeptides, respectively (Figure 3F).

### 2.6. Properties of Phosphorylated Proteins

**Figure 4:**
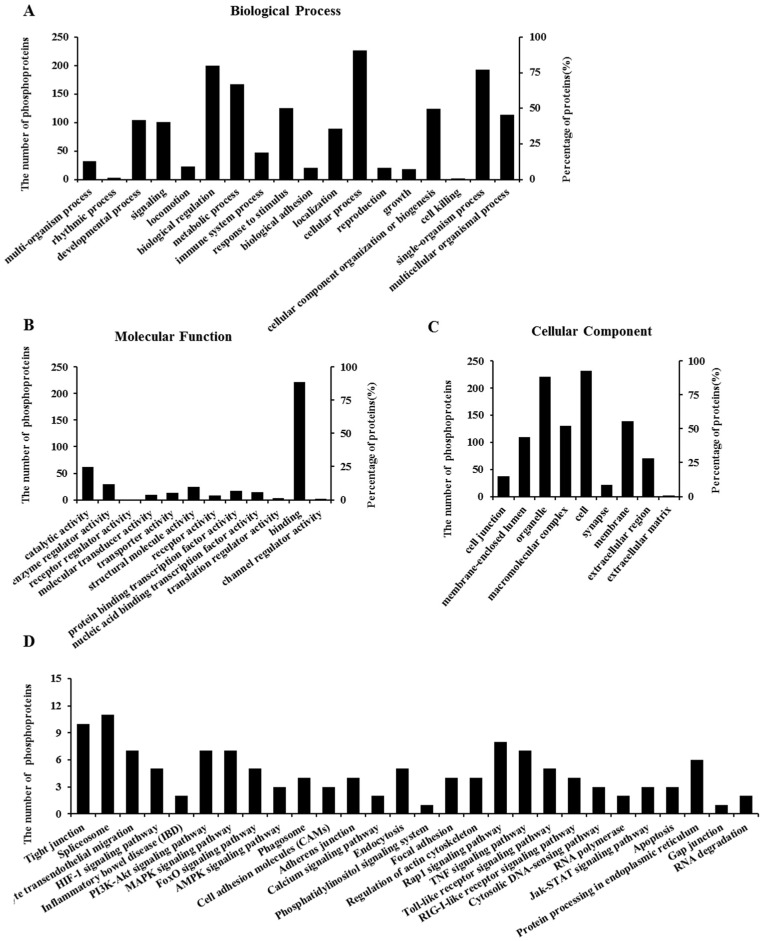
Gene Ontology (GO) and Kyoto Encyclopedia of Genes and Genomes (KEGG) pathway enrichment analysis of differentially regulated phosphoproteins after DON exposure in differentiated IPEC-J2 cells. Fold-change > ±1.2. Data are presented according to the (**A**) biological process; (**B**) molecular function; and (**C**) cellular component (or sub-cellular localization) and shown as a percentage of the total number of differentially identified phosphoproteins (255) that fall within each category; (**D**) Data are presented according to the differentially identified phosphoproteins (255) involved in the relevant signaling pathways, which were classified into 29 different categories depending on their predicted functions.

To obtain insights into the function of DON-responsive phosphoproteins, we performed a Gene Ontology (GO) annotation and enrichment analysis for differentially phosphorylated proteins. As shown in Figure 4, these differentially expressed phosphoproteins were classified into 18 groups according to the properties of their biological process (Figure 4A), 12 groups according to their molecular functions (Figure 4B), and nine groups according to their cellular components (Figure 4C). 

To obtain a further general overview role of the 255 differentially phosphoproteins affected by DON, Kyoto Encyclopedia of Genes and Genomes (KEGG) pathway enrichment analysis was employed. As shown in Figure 4D, 29 primary different categories were defined according to their predicted functions, covering a wide range of pathways. The primary functional groups were tight junction, leukocyte transendothelial migration, inflammatory bowel disease (IBD), MAPKs, forkhead box O (FoxO), phosphatidylinositol 3-kinase/Akt (PI3K/Akt), Janus kinase/signal transducer, and activator of transcription (JAK/STAT) pathways, as well as apoptosis. Detailed information can be found in Table S4. In addition, we have listed the crucial phosphorylated proteins associated with the above functional categories as well as their corresponding phosphorylation sites (Tables S4 and S5).

The hierarchical clustering of these differentially expressed phosphoproteins is visualized in a heat map (Figure S1). This data model shows high similarities within groups and lower similarity between the groups. Thus, the results of target phosphoprotein screening were satisfactory, and phosphorylation changes correspond to significant impact of biological replicates.

### 2.7. Validation of Selected Proteins

**Figure 5:**
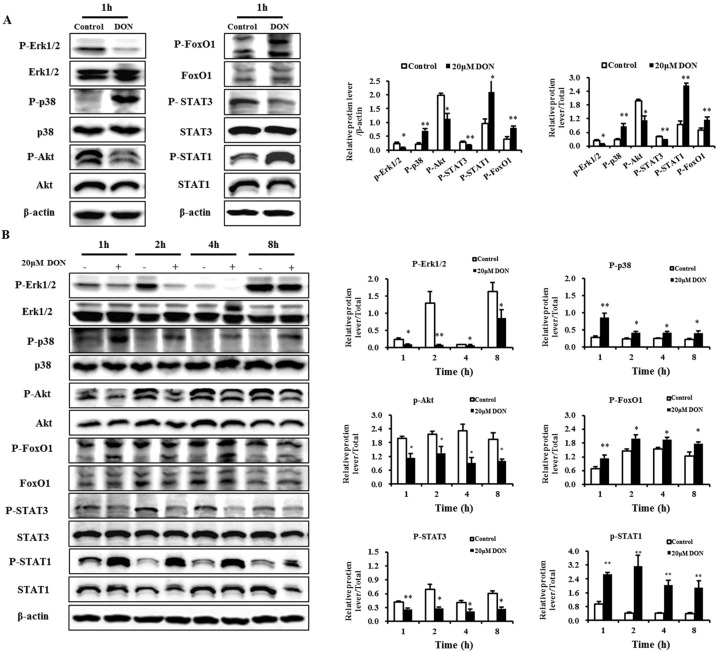
Activities of Erk1/2, p38, Akt, FoxO1, STAT3, and STAT1 in differentiated IPEC-J2 cells exposed to DON. (**A**) Activities of Erk1/2, p38, Akt, FoxO1, STAT3, and STAT1 in the differentiated IPEC-J2 cells (on day 7) treated for 1 h with 20 μM DON; (**B**) Activities of Erk1/2, p38, Akt, FoxO1, STAT3, and STAT1 in differentiated IPEC-J2 cells (day 7) treated for 1 h, 2 h, 4 h, and 8 h with 20 μM DON. After the proteins were isolated, the total and phosphorylation-specific antibody reactions were assessed using Western blotting, with total protein or β-actin as a loading control. All results were obtained using six replicates (*n* = 6), and data are presented as means ± SEM (standard error of the mean). *, *p* ≤ 0.05; **, *p* ≤ 0.01.

To verify phosphorylation quantification obtained using iTRAQ-based phosphorproteomics, phosphorylation of p38 and STAT1 was measured using Western blotting. The results demonstrated that DON significantly increased the phosphorylation of p38 (Thr180/Tyr182) and STAT1 (Ser727) after 1 h (Figure 5A), and that during a longer period (1–8 h) the same tendency was observed (Figure 5B). Thus, iTRAQ- and immunoblot-based relative phosphorylation quantification and the identification of phosphorylation sites using these two methods derive similar results. We demonstrated that iTRAQ combined with the light chromatography (LC)-MS/MS method is a viable approach to study the DON-induced intestinal toxicity.

Our results also showed that DON significantly inhibited the phosphorylation of Erk1/2 and Akt at 1 h, 2 h, 4 h and 8 h time points (Figure 5). The results presented here provide new evidence of association of p38 and Erk1/2 MAPKs with DON-induced intestinal toxicity. We also examined the phosphorylation of the two representative transcriptional regulation factors, FoxO1 and STAT3, which belong to the FoxO and JAK/STAT pathways. Results showed that 20 μM DON significantly increased the phosphorylation of FoxO1 at all time points (Figure 5), indicating that DON induced the nuclear exclusion of FoxO1. We also found that 20 μM DON significantly decreased the levels of phospho-STAT3 (Figure 5). The results presented here confirm the association of FoxO, PI3K/Akt and the JAK/STAT pathway with DON-induced intestinal toxicity. However, the iTRAQ-based phosphoproteomic study failed to detect the phosphorylation of Erk1/2, Akt, FoxO1, and STAT3. It is likely that during the sample isolation, fractionation, and digestion procedures some proteins might have been removed, resulting in reduced sensitivity of the method.

## 3. Discussion

**Figure 6:**
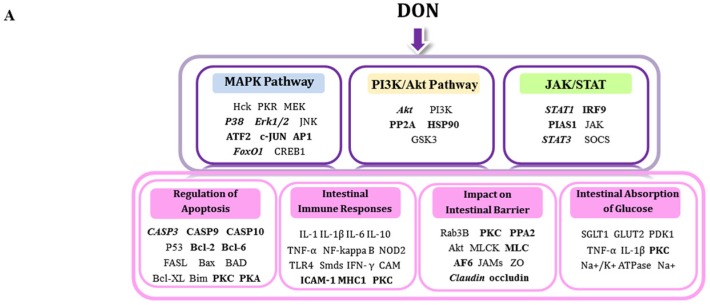

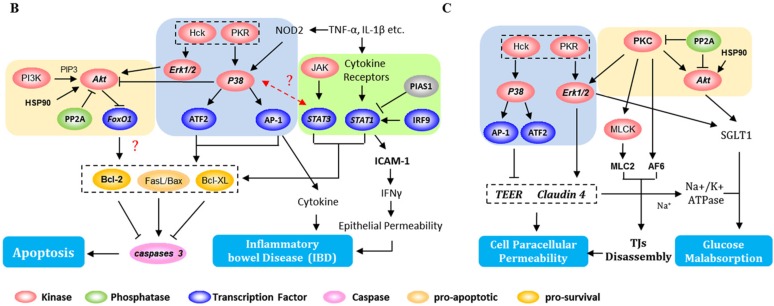
Summary of potential signaling pathways and biological processes involved in the DON-induced intestinal toxicity in differentiated IPEC-J2 cells. (**A**) Signaling pathways affected included MAPKs (blue), PI3K/Akt (orange), and JAK/STAT (green); The key biological processes included regulation of (**B**) apoptosis, intestinal immune responses; (**C**) intestinal barrier, and intestinal absorption of glucose. The bold font indicates the novel mediators identified in this study, the bold and italics denote the mediators verified by Western blot, and normal font denotes the previously known mediators that are not identified here.

As the most frequently encountered mycotoxins, the contaminative level of DON in cereal-based foods is up to approximately 1.2–19 mg/g [2,3,25]. Since DON is a daily food contaminant, as prominent barrier for nutritional toxins [10], the intestine has to regularly handle a high concentration of DON from the luminal side. Since the concentrations of DON treatment to IECs was normally adopted in higher μM range [12,26,27,28,29,30], it appears that sensitivity of intestine is very low (1 µM to 100 µM) [31]. Studies have confirmed that apical application of DON up to 6.75–13.5 μM has no significant effect on TEER and expression of claudin-3, claudin-4 or ZO-1 [12,27]. Consistent with previous studies, we observed that 10 μM DON treatment caused a tendency to reduce TEER. However, after treatment with 20 μM DON over a 24 h period, TEER and claudin-4 significantly decreased. In addition, an increase in levels of the cleaved form of caspase-3 and a significant inhibitory effect on the glucose consumption were also found. Therefore, to obtain a thorough molecular perspective on the signaling associated with key biological processes affected by a high luminal DON challenge of IECs, this study constructed a large-scale quantitative dataset of phosphorylation changes in IPEC-J2 cells in response to 20 μM DON from apical side, using an iTRAQ-based phosphoproteomic approach. Moreover, 20 μM DON corresponds to 6 mg/kg in food and encompasses levels that previously were observed in cereal-based food or feed. Apart from the well-known MAPK signaling proteins, our data revealed an array of phosphoproteins with several candidate phosphorylation sites. These proteins are associated with PI3K/Akt and JAK/STAT pathways, which are involved in regulating the apoptosis, intestinal barrier, intestinal inflammation, and the intestinal absorption of glucose (Figure 6A).

### 3.1. DON-Induced Changes—Impact on Apoptosis

DON-induced apoptosis, already demonstrated in various cells, might seriously inhibit immune function [32,33]. DON treatment also evokes apoptosis in colon carcinoma cells and intestinal epithelial cell lines [34,35]. Consistent with previous studies, we found in the IPEC-J2 cells exposed to DON, an increase in levels of the cleaved form of caspase-3, a critical executioner of apoptosis [36]. We also found that DON-induced apoptosis was associated with caspase-3 activation. However, Diesing et al. showed no caspase-3 activation after lower DON exposure (24 h) from apical or basolateral side [27]. This may be related to low dose effects of DON on IECs being triggered by mechanisms different from those responsible for the high dose toxicity [13]. It is worth noting that DON activates the apoptotic (p38/p53/caspase-3) and survival (Erk1/2/Akt/Bad) pathways in RAW264.7 macrophages [32]. ERKs enhance the activation of Akt [37], while p38 might be involved in its inactivation [38]. In addition, the phosphatase 2A (PP2A) can dephosphorylate Akt, making it inactive, whereas heat shock protein 90 (Hsp90) enhances its activity [39,40]. Once activated, Akt phosphorylates caspase-9 directly and inhibits its protease activity [41], functioning as a robust intracellular prosurvival signal [32]. Although we provide no direct evidence of Akt regulation, we identified a significant decrease in Erk1/2 phosphorylation and an increase in the phosphorylation of p38 (Thr180/Tyr182). We also observed an increase in the phosphorylation of PP2A (Thr130/Ser146), a decrease in the phosphorylation of Hsp90 (Ser263/231), and a particularly noteworthy reduction in the phosphorylation of caspase-9 (Ser302). All these results suggested that DON inhibited Akt. FoxO1 is a downstream target of Akt, and its nuclear exclusion results in the reduction of transcriptional activity of the pro-apoptotic Bcl-2 family [42]. Nevertheless, we found increased phosphorylation of FoxO1, which might activate the survival pathway. Apart from Akt, the activating transcription factor-2 (ATF-2) and activator protein 1 (AP1) are also targets of the p38-MAPK-regulated apoptosis [43,44]. We also observed an increase in the phosphorylation of ATF-2 (Ser90/Thr69/71) and AP1 (Ser73/Thr62), as well as a decrease in Bcl-2 phosphorylation (Ser133). Thus, it seemed that Akt might be located downstream of the p38/Erk1/2 signaling pathway, with p38 playing a critical role as the pro-apoptotic factor. Additionally, the observed reduction in the phosphorylation of Erk1/2 might enhance the pro-apoptotic effects of p38.

A recent study has reported that the JAK/STAT pathway plays a pivotal role in the apoptosis induced by DON in RAW264.7 cells [45]. STAT1 plays a vital role in stimulating apoptosis [46], whereas STAT3 inhibits it by promoting the anti-apoptotic gene expression [47]. We observed increased phosphorylation of STAT1 (Ser727) and the loss of STAT3 phosphorylation, illustrating the different facets of pro-apoptotic activity induced by DON. Although interferon regulatory factor 9 (IRF9) might be important for the transactivation activity of STAT proteins [48], protein inhibitors of activated STAT1 (PIAS1) suppress its transcriptional activity [49]. Hence, this hypothesis was further confirmed by the increased phosphorylation of IRF9 (Ser63) as well as by a loss of phosphorylation on PIAS1 (Thr460/Ser468). Furthermore, because activation of JAK/STAT induced by DON occurs much later than the activation of MAPKs in RAW264.7 cells, it has been suggested that STAT3 activation is dependent on p38 [45]. Therefore, we speculated that STAT1/STAT3 might be the downstream targets of MAPKs, and there might be a potential crosstalk between MAPKs and JAK/STAT pathways. However, further studies are required to validate this hypothesis.

### 3.2. DON-Induced Changes-Impact on Intestinal Barrier

IECs are tightly packed with intercellular junction complexes that regulate paracellular permeability and integrity of the epithelial barrier [50]. Dysfunction of the intestinal barrier is associated with increased gut permeability accompanied by the development of various gastrointestinal diseases [51]. Some studies have indicated that DON dramatically alters barrier function and intestinal permeability via modulation of the tight junctions (TJs) or mucus layer [12,14,52]. In agreement with previous studies, we observed that, after DON exposure, TEER and claudin-4 expression decreased significantly. Intestinal barrier functions are regulated by phosphorylation, involving different types of signaling proteins, including MAPKs, protein kinase C (PKC), and myosin light chain kinase (MLCK) [53]. MAPKs are involved in impaired intestinal barrier function, and the expression of several tight junction proteins and mucus secretion might be affected by DON [7,12,54,55]. We identified a significant decrease in Erk1/2 phosphorylation, and an increase in p38 phosphorylation. Hence, we suggest that the decrease in TEER and claudin-4 expression after DON exposure might be associated with p38 activation and subsequent Erk1/2 inactivation. And probablely, DON is able to affect the expression and production of mucins through activation of p38 pathways. There is some evidence that an increase in TJ levels is Erk1/2-dependent [56]. However, other studies have demonstrated that the activation of Erk1/2 by DON results in an increase in the paracellular permeability in IPEC-1 cells [12,57]. On one hand, this difference may be associated with different types of cells. Nossol et al. showed that IPEC-J2 is a morphologically and functionally more differentiated cell line in comparison to IPEC-1 [24]. It is worth noting that “tight junction” pathways are significantly different in the two cell lines. Therefore, DON may impair intestinal barrier functions through different mechanisms in different cells. On the other hand, probably it is not the leading role by Erk1/2 in regulating barrier functions of DON in IPEC-J2 cells. Consistent with the previous study [27], we identified a significant decrease in Erk1/2 phosphorylation, which might enhance the pro-apoptotic effects of DON. What is more, besides Erk1/2, intestinal barrier functions may be regulated by other different types of signaling proteins. As the role of Erk1/2 in the intestinal barrier functions regulation by DON is still controversial, further research is needed to clarify its mechanisms. Apart from the MAPKs, the PKC also affects barrier integrity through TJ modulation [58], whereas PP2A directly dephosphorylates the PKC involved in the TJs’ disassembly [59]. Furthermore, phosphorylation of myosin light chain 2 (MLC2) by MLCK is associated with TJ disassembly [60]. Crosstalk between PKC and MLCK appears to be involved in the regulation of TJs’ assembly and disassembly. PKC activation is always accompanied by a progressive decrease in MLC2 phosphorylation [61]. The results presented here demonstrated that DON caused a significant increase in PP2A phosphorylation and decrease in PKC phosphorylation (Thr218), followed by upregulation of MLC2 phosphorylation (Ser1863). Hence, we propose that DON might provoke the disassembly of TJs, leading to an increase in the paracellular permeability via crosstalk between PKC and MLCK. As a component of adherens junctions, afadin-6 (AF-6) is heavily involved in the recruitment and binding of various junctional proteins, including junctional adhesion molecules [62]. Because we observed a significant decrease in the phosphorylation of AF-6 (Ser1274) following DON exposure, we might infer that AF-6 disturbs assembly interactions and the dynamic modulation of cell-cell junctions.

### 3.3. DON-Induced Changes—Impact on Intestinal Immune Responses

The intestine is the first barrier against food contaminants and plays a pivotal role in local and systemic immune responses [63]. Consistent with their known pro-inflammatory activities, cytokines and chemokines cause a disturbance in the intestinal barrier, leading directly to an increase in gut permeability, which might result in IBD [64]. The molecular features associated with IBD are similar to the characteristic changes observed in intestinal cells after DON exposure [65]. For example, the nucleotide-binding oligomerization domain-containing protein 2 (NOD2) is a critical regulator associated with gastrointestinal immunity. It causes activation of MAPKs and is involved in pro-inflammatory responses [66]. After DON exposure, we observed a significant increase in the phosphorylation of p38 and the downstream target AP-1, which might be a consequence of NOD2→p38→AP1 signaling activation contributing to the induction of intestinal mucosal inflammation by DON. In addition to AP-1, we found increased phosphorylation of STAT1 and STAT3, the crucial transcription factors involved in Th1 and Th17 cell differentiation, respectively [67]. Recent studies have suggested that DON mainly drives the intestinal immune system towards a Th17 response [68]; however, we observed a significant increase in the phosphorylation of STAT1, but a decrease in STAT3 phosphorylation. As a result, we tentatively put forward that DON mainly initiates the intestinal immune response elicited by Th1 rather than the Th17 response. Nevertheless, the suggestive Th1 response elicited by DON requires further study. In addition to the cytokines, STAT1 has been recently shown to associate with the activation of expression of intercellular adhesion molecule-1 (ICAM-1), a signal transduction molecule regulating the transepithelial migration of polymorphonuclear cells (PMN). Such activation might result in an increased epithelial permeability associated with the pathogenesis of IBD [69]. In this study, we identified an increase in the phosphorylation of ICAM-1 (Ser535), and therefore speculated that it might have impaired the epithelial barrier, increasing the numbers of infiltrating PMNs, which might ultimately lead to mucosal injury.

### 3.4. DON-Induced Changes—Impact on the Absorption of Glucose

Nutrient malabsorption can be caused by several adverse factors that alter the gastrointestinal barrier. Indeed, some experiments have shown that DON inhibits the intestinal absorption of glucose in various species [16,26]. In agreement with these studies, we also found that DON had a significant inhibitory effect on the glucose consumption in IPEC-J2 cells. However, relatively little is known about the toxicity mechanism underlying the effect of DON on glucose malabsorption. The classic pattern of intestinal glucose absorption is that luminal glucose is transported into enterocytes by the Na^+^/glucose cotransporter sodium-glucose cotransporter 1 (SGLT1), and by the facilitated- diffusion glucose transporter glucose transporter 2 (GLUT2) into the portal venous system [70]. It has been shown that GLUT2 is slightly inhibited by DON [26], and as expected, SGLT1 is the dominant DON-sensitive transporter in the IECs of poultry [71]. Some researchers have proposed that DON decreases the levels of SGLT-1 by inhibiting protein synthesis [26]. However, DON treatment reduces glucose uptake almost as efficiently as phlorizin, a specific inhibitor of SGLT-1 [16]. The activity of SGLT-1 might be regulated by PKC via the downstream signal cascades of MAPKs and PI3K/Akt, through the activation or inactivation mechanisms associated with glucose malabsorption [72]. Our data showed that DON significantly decreased the phosphorylation of PKC, Akt, and Erk1/2. An increase in the phosphorylation of PP2A and a decrease in the phosphorylation of Hsp90 were also observed. These proteins are the negative and positive regulatory elements of Akt, respectively. Thus, we suggest that, in IPEC-J2 cells, DON exposure lowers the activity of SGLT1 via PKC inactivation with Erk1/2 or Akt as downstream targets, resulting in a reduction in glucose transport. Apart from the involved intracellular signal cascades, the paracellular transport of Na^+^ is thought to be critical for nutrient absorption by Na^+^-driven transporters including SGLT1 [73], and some recent studies have reported that TJs are indispensable for transepithelial paracellular permeability to extracellular Na^+^ [74,75]. Impairment of the Na^+^ gradient across the intestinal barrier might be associated with modification of Na^+^/K^+^-ATPase activity. Here, we observed that both TEER and claudin-4 expression were significantly reduced. Therefore, glucose malabsorption caused by DON can be ascribed to the breakdown of the Na^+^ gradient, and the activity of Na^+^/K^+^-ATPase was significantly diminished.

In a word, our results suggest that four distinct events might occur after IPEC-J2 cell exposure to DON. (1) Apoptotic pathways, including p38→ATF-2/AP1, and STAT1/STAT3 are activated whereas the Erk1/2→Akt survival pathway is inhibited. This might be followed by Bcl-2 protein downregulation and caspase-3 activation (Figure 6B); (2) p38/Erk1/2 alters the expression of claudin-4 and TEER, which increases paracellular permeability. This effect is reinforced by PKC/MLC2 interactions and a decrease in AF-6 phosphorylation, which might affect the TJ assembly (Figure 6C); (3) p38→AP1 or STAT1→cytokine pathways might be triggered, leading to intestinal inflammation. The STAT1→ICAM-1 system is associated with the response to bacterial infections. They all toward a Th1 response, that is likely to be associated with the pathogenesis of IBD (Figure 6B); (4) SGLT-1 activity might be regulated by PKC, affecting the downstream signal cascades MAPKs and PI3K/Akt. Impairment of the Na^+^ gradient across the intestinal paracellular barrier might occur as a result of TJ protein downregulation, and glucose malabsorption follows (Figure 6C).

## 4. Conclusions

The global, quantitative phosphoproteomic analysis presented here contributes to our knowledge of critical signaling events involved in deoxynivalenol (DON)-initiated intestinal toxicity. There are several potential participants in this process, ranging from MAPKs to the complex signaling network involving the PI3K/Akt and JAK/STAT cascades (Figure 6). This is the first study showing that DON exposure alters the phosphorylation states and sites of multiple proteins found in differentiated intestinal epithelial cells (IECs). Our study also clarifies some of the molecular mechanisms underlying the toxic effects of DON, and provides a database resource including previously unknown phosphorproteins and phosphorylation sites for the future exploration of the signaling pathways involved in the intestinal toxicity evoked by DON. Our findings might help in the formulation of effective therapeutic strategies for the treatment of intestinal dysfunction. 

## 5. Materials and Methods

### 5.1. Cell Culture and Reagents

The IPEC-J2 cell lines used in this study were a gift from Daiwen Chen (College of Animal Science and Technology, Sichuan Agricultural University, Ya’an, China). The cells were cultured in Dulbecco’s Modified Eagle Medium (DMEM/Ham’s F-12 [1:1]) supplemented with 5% fetal bovine serum (FBS), antibiotics (penicillin sodium salt and streptomycin sulfate), 1% Insulin-Transferrin Selenium (Life-Technologies, Stoughton, MA, USA) and 5 ng/mL of epidermal growth factor (Sigma-Aldrich, St. Louis, MO, USA), incubated at 37 °C and in 5% CO_2_. The IPEC-J2 cells between passages 70–80 were grown and differentiated as previously described [76]. The differentiation medium used was as described above but FBS was substituted with 10^−7^ M dexamethasone (Sigma-Aldrich). Cells showed features of differentiated cells as judged by a significant increase in TEER (TEER value > 1 kΩ/well) [77]. The apical compartments of the inserts were used to introduce the treatment compound, and purified DON (Sigma-Aldrich) was dissolved in double distilled water and stored at −20 °C before dilution in the cell culture medium.

### 5.2. DON Exposure

IPEC-J2 cells were seeded at 3 × 10^5^ cells/well in 4.67 cm^2^ Transwell^®^ Permeable Supports (0.4-μm pore size) (Corning Inc., Corning, NY, USA), reaching confluence within two days. Subsequently, the differentiation medium was applied and changed every other day, and when fully differentiated, cells were treated with chosen concentrations of DON in the apical compartment at different time points. Transepithelial electrical resistance (TEER) measurements, claudin-4 and caspase-3 expression detection, glucose consumption tests, phosphorproteome analysis, and signaling pathway protein verification were performed.

### 5.3. Measurement of Transepithelial Electrical Resistance (TEER)

TEER was measured during the 16 culture days using a Millicell-ERS 2 Voltohmmeter (Millipore, Billerica, MA, USA). When cells were fully differentiated, TEER (treated with 10, or 20 μM DON for 4 h, 8 h, 12 h, and 24 h in six wells) was measured. TEER values are given as kΩ/well (membrane area 4.67 cm^2^) with 1 kΩ being the level of confluence. 

### 5.4. Glucose Assay

Glucose measurement was performed on samples of medium. At the appropriate stage of differentiation, cell supernatant (treated with 20 μM DON for 1 h, 2 h, 4 h, 8 h, 16 h and 24 h in six wells) from the apical side was collected, and remaining glucose levels were measured using an enzymatic assay based on the method of Trinder [78]. Data were normalized using the corresponding cellular protein content.

### 5.5. Protein Analyses

#### 5.5.1. Sample Preparation

For Western blot analysis, proteins were extracted from cells (treated with 20 μM DON for 1 h, 2 h, 4 h, 8 h, 16 h and 24 h in six wells) and quantified as previously described [74]. Proteins were extracted with radio immune-precipitation assay lysis buffer (50 mM Tris, 150 mM NaCl, 1% Triton X-100, 1% sodium deoxycholate, 0.1% SDS, pH 7.4) (Beyotime, Shanghai, China) containing protease inhibitor cocktail (Roche, Basel, Switzerland). For phosphoproteome analysis, moreover, cells exposed to 20 μM DON for 1 h were homogenized in the extraction buffer (4% SDS, 100 mM Tris-HCl, 1 mM·DTT, pH 7.6). The homogenate was then sonicated after boiling in the water bath for 3 min, and the protein extracts were centrifuged at 13,400 rpm for 30 min. Finally, the protein concentration was determined using bicinchoninic acid (BCA) protein assay reagent kit (Pierce, Rockford, IL, USA).

#### 5.5.2. Western Blot Analysis

Proteins were extracted from cells (treated with 20 μM DON for 1 h, 2 h, 4 h and 8 h in six wells) and quantified as previously described [79]. After degenerating, 30 μg of protein were separated by electrophoresis at 300 V for 20 min, and proteins were subsequently electrotransferred onto polyvinylidene difluoride membranes (Millipore). The membranes were blocked for 1.5 h using 5% (*v*/*v*) non-fat milk at room temperature and then incubated overnight at 4 °C with the antibodies diluted 1:1000) of: anti-claudin-4 (Invitrogen, Carlsbad, CA, USA), β-actin, p38, phospho-p38 (Thr180/Tyr182), Erk1/2, phospho-Erk1/2 (Thr202/Tyr204), FoxO1, phospho-FoxO1 (Ser256), STAT3, phospho-STAT3 (Tyr705), STAT1, phospho-STAT1 (Ser727), Akt, phospho-Akt (Ser473), caspase-3, cleave caspase-3 (Asp175) antibodies (Cell Signaling, Danvers, MA, USA). Anti-β-actin antibody was used as loading control. Membranes were then washed (five times, 3 min each wash). This was followed by a 1 h incubation with horseradish peroxidase-labeled goat anti-mouse/chicken/rabbit IgG (Santa Cruz Biotechnology, Dallas, TX, USA) at a dilution of 1:1000. Finally, chemiluminescent bands were visualized on the membranes using ChemiDoc™ Touch Imaging System (Bio-Rad, Hercules, CA, USA).

#### 5.5.3. Protein Digestion, iTRAQ Labeling and Phosphopeptide Enrichment

Protein digestion was performed following the FASP procedure as reported previously [80], and the peptide mixture obtained was labeled using the eight-plex iTRAQ reagent according to the manufacturer’s directions (Applied Biosystems, Foster City, CA, USA). Briefly, 400 μg of protein from each sample was added to DTT at a final concentration of 100 mM. The samples were boiled in a water bath for 5 min, cooled to room temperature, and mixed with uric acid (UA) buffer (8 M urea, 150 mM Tris-HCl pH 8.0) by repeated ultrafiltration. Then, 100 mM iodoacetamide in UA buffer was added, and samples were incubated for 30 min in darkness. The filtrates were washed three times with 100 μL of UA buffer and then twice with 100 μL of the dissolution buffer (DS). Finally, protein suspensions were digested with 4 μg of trypsin (Promega, Madison, WI, USA) in 40 μL of DS buffer at 37 °C overnight. After desalting and concentration, the peptide content was estimated by measuring spectral density at 280 nm. Approximately 200 μg of the digested protein samples from the two groups were labeled as (C1)-113, (C2)-114, (C3)-115, (T1)-116, (T2)-117, (T3)-118, and were multiplexed and vacuum dried. Three biological replicates were used.

The iTRAQ-labeled peptides were resuspended in 500 μL of 1×DHB loading buffer (3% DHB, 80% ACN, 0.1% TFA). Then TiO2 beads were added and agitated for 40 min. After centrifugation, the supernatant was removed, and the pellet washed with 50 μL of washing buffer I (30% ACN, 3% TFA) and then with 50 μL of washing buffer II (80% ACN, 0.3% TFA) three times in both cases. Finally, phosphopeptides were eluted with 50 μL of elution buffer (40% ACN, 15% NH_4_OH), vacuum-concentrated and reconstituted in 20 μL of 0.1% (*v*/*v*) formic acid (FA). The samples were stored at −80 °C prior to LC-MS/MS analysis.

### 5.6. Liquid Chromatography-Tandem Mass Spectromety (LC-MS/MS) Analysis

LC-MS/MS analysis was performed using a Q Exactive Mass Spectrometer coupled to Easy-nLC HPLC system (Thermo Fisher Scientific, San Jose, CA, USA). Peptides were first trapped onto a C18 pre-column (Thermo Scientific Acclaim PepMap100, 100 μm × 2 cm, nanoViper C18), before being separated on an analytical column (Thermo Scientific Easy Column, 10 cm long, 75 μm inner diameter, 3 μm resin) in buffer A (0.1% FA) and eluted with a linear gradient of buffer B (84% acetonitrile and 0.1% FA) at a flow rate of 300 nL/min. We used a 4 h gradient: 0%–55% buffer B for 220 min, 55%–100% buffer B for 8 min, and a hold in 100% buffer B for 12 min. The mass spectrometer was operated in a positive ion mode, and analysis lasted 240 min. Survey scan MS spectra (350–1800 *m*/*z*) were acquired with an automatic gain control target of 3e6, and a maximum injection time of 20 ms. A dynamic exclusion with an exclusion duration of 30 s was applied. Survey scans were acquired at a resolution of 70,000 at *m*/*z* 200 and the resolution for higher energy C-trap dissociation (HCD) spectra was set to 17,500 at *m*/*z* 200. The normalized collision energy was 29 eV.

### 5.7. Data Analysis

Database was searched using the MASCOT engine (Version 2.2, Matrix Science, London, UK) embedded in the Proteome Discoverer 1.4 (Thermo Fisher Scientific) against the UniProt *Sus scrofa* database (35,143 sequences, downloaded on 21 August 2015). Parameters were set as follows: peptide mass tolerance = 20 ppm; MS/MS tolerance = 0.1 Da, enzyme = trypsin, missed cleavage = 2, fixed modification: carbamidomethyl (C), iTRAQ4/8plex (N-term), iTRAQ 4/8plex(K), carbamidomethyl (C), variable modification: oxidation (M), iTRAQ four/eight-plex (Y), phosphorylation (S/T/Y), peptide FDR ≤ 0.01, significant difference analysis: phosphorylated modifications fold-change > ±1.2.

### 5.8. Bioinformatic Analysis of Phosphoproteomic Data

Differentially expressed proteins of the phosphoproteome were retrieved from the UniProtKB database (Release 2015_10) in FASTA format. Retrieved sequences were locally searched against the Swiss-Prot database (*Sus scrofa*) using the NCBI BLAST + client software (ncbi-blast-2.2.28+- win32.exe, Version 2.2.28+, NCBI, MT, USA). GO mapping and annotation, KEGG orthology identifications and subsequent mapping to pathways, in addition to enrichment analysis of GO or KEGG pathway annotations were carried out as described by Zhu et al. [81]. Finally, the capability of the differentially expressed proteins identified was evaluated using hierarchical clustering analysis (Version 3.0, Stanford University, Stafford, CA, USA).

### 5.9. Statistical Analysis

All data about TEER, glucose assay and Western blot analysis are expressed as means ± standard error of the mean (SEM). Data obeyed normal distribution, and variance between the control and treatment group was determined by Student’s *t*-test; one-way ANOVA were performed for comparison between data from cells treated with different doses of DON (SPSS 18.0, IBM Inc., Armonk, NY, USA), and significant differences were set at *, *p* ≤ 0.05 and **, *p* ≤ 0.01.

## Supplementary Materials

The following are available online at www.mdpi.com/2072-6651/8/10/270/s1. Figure S1: Cluster heat map of differentially expressed phosphoproteins regulated by DON exposure in differentiated IPEC-J2 cells. Each column represents a group from three biological replicates (C: Control; T: 20 μM DON). The color codes indicate the average values of the biological replicates. Table S1: Identification of phosphopeptides in differentiated IPEC-J2 cells after DON exposure. The sequence data of the phosphoproteome are shown in groups retrieved from the UniProtKB database (uniprot_SUS_SCROFA_35143_20150821.fasta) in FASTA format. Table S2: Characterization of differentially expressed phosphopeptides in differentiated IPEC-J2 cells after DON exposure. There were 289phosphopeptides differentially regulated after DON exposure as determined by a fold-change > ±1.2. Table S3: Identification of phosphoproteins and differentially expressed phosphoproteins in differentiated IPEC-J2 cells after DON exposure. For 4234 unique phosphopeptides, 1821 phosphoproteins were identified. Phosphoproteins were considered differentially expressed when the fold-change was >±1.2. On the basis of this criterion, 255 differentially phosphoproteins were identified. Table S4: Primary pathways associated with differentially expressed phosphoproteins affected by DON exposure in differentiated IPEC-J2 cells. Table S5: Phosphorylated proteins associated with the key functional categories induced by DON and their corresponding phosphorylation sites.
