# Supplementary material for: Phosphoproteome Analysis Reveals the Molecular Mechanisms Underlying Deoxynivalenol-Induced Intestinal Toxicity in IPEC-J2 Cells
Source: Toxins (Basel). 2016 Sep 22;8(10):270. doi: 10.3390/toxins8100270 (PMC5086631; doi:10.3390/toxins8100270)
Supplement: Supplementary file 1 [file toxins-08-00270-s001.zip › toxins-144180-supplementary/toxins-144180-figure-supplementary-checked.pdf]

Supplementary Materials: Phosphoproteome Analysis Reveals the Molecular Mechanisms Underlying Deoxynivalenol-Induced Intestinal Toxicity in IPEC-J2 Cells

Zhi-Qi Zhang, Song-Bo Wang, Rui-Guo Wang, Wei Zhang, Pei-Long Wang and Xiao-Ou Su

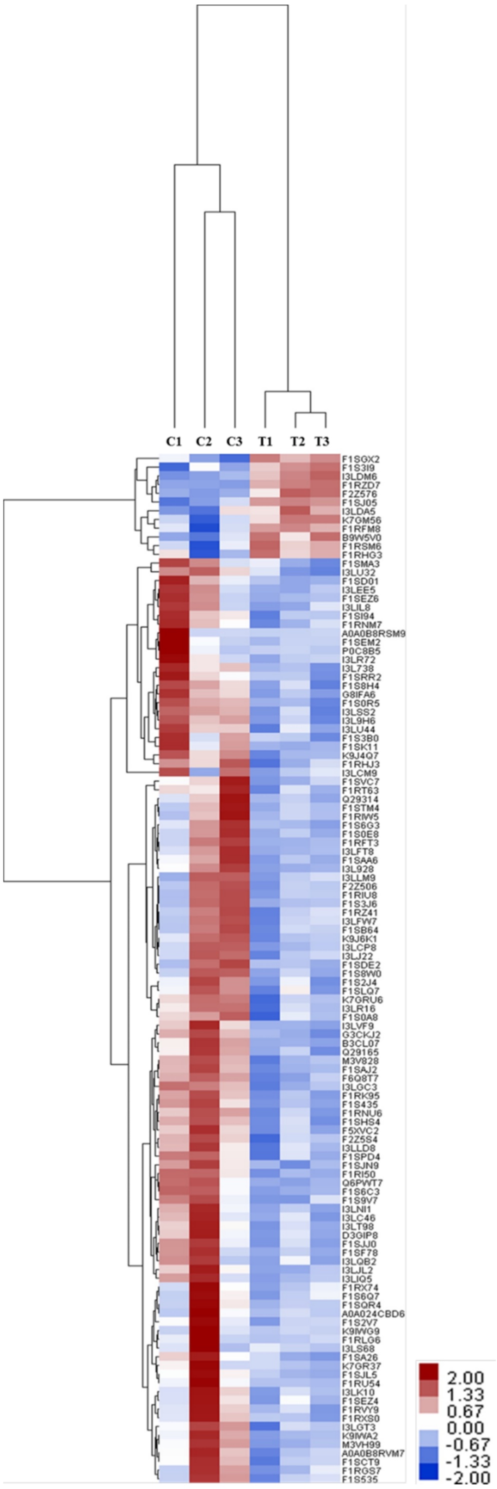

Figure S1. Cluster heat map of differentially expressed phosphoproteins regulated by DON exposure in differentiated IPEC-J2 cells. Each column represents a group from three biological replicates (C: Control; T: 20 μM DON). The color codes indicate the average values of the biological replicates.
